# Vaccine Platform Comparison: Protective Efficacy against Lethal Marburg Virus Challenge in the Hamster Model

**DOI:** 10.3390/ijms25158516

**Published:** 2024-08-05

**Authors:** Kyle L. O’Donnell, Corey W. Henderson, Hanna Anhalt, Joan Fusco, Jesse H. Erasmus, Teresa Lambe, Andrea Marzi

**Affiliations:** 1 Laboratory of Virology, Division of Intramural Research, National Institute of Allergy and Infectious Diseases, National Institutes of Health, Hamilton, MT 59840, USA; 2 Public Health Vaccines Inc., Cambridge, MA 02412, USA; 3 HDT Bio, Seattle, WA 98102, USA; 4 Oxford Vaccine Group, Department of Paediatrics, University of Oxford, Oxford OX3 7BN, UK

**Keywords:** MARV, vesicular stomatitis virus, VSV, ChAdOx, replicating RNA, repRNA/LION, antibody, Fc effector function

## Abstract

Marburg virus (MARV), a filovirus, was first identified in 1967 in Marburg, Germany, and Belgrade, former Yugoslavia. Since then, MARV has caused sporadic outbreaks of human disease with high case fatality rates in parts of Africa, with the largest outbreak occurring in 2004/05 in Angola. From 2021 to 2023, MARV outbreaks occurred in Guinea, Ghana, New Guinea, and Tanzania, emphasizing the expansion of its endemic area into new geographical regions. There are currently no approved vaccines or therapeutics targeting MARV, but several vaccine candidates have shown promise in preclinical studies. We compared three vaccine platforms simultaneously by vaccinating hamsters with either a single dose of an adenovirus-based (ChAdOx-1 MARV) vaccine, an alphavirus replicon-based RNA (LION-MARV) vaccine, or a recombinant vesicular stomatitis virus-based (VSV-MARV) vaccine, all expressing the MARV glycoprotein as the antigen. Lethal challenge with hamster-adapted MARV 4 weeks after vaccination resulted in uniform protection of the VSV-MARV and LION-MARV groups and 83% of the ChAdOx-1 MARV group. Assessment of the antigen-specific humoral response and its functionality revealed vaccine-platform-dependent differences, particularly in the Fc effector functions.

## 1. Introduction

Marburg virus (MARV) is a member of the *Filoviridae*, the same family as Ebola virus (EBOV), and has a 19 kb (-) single-stranded RNA genome encoding seven proteins. The mature viral particles are filamentous in structure and exit the cell through budding from the surface [1]. MARV was first identified in 1967 in Marburg, Germany, and Belgrade, former Yugoslavia, and from then on it has caused sporadic outbreaks of human Marburg virus disease (MVD) in parts of Africa [2,3]. The largest outbreak occurred in 2004–2005 in Angola, where 252 cases were identified and 227 fatalities were recorded [4]. The 2021–2022 outbreaks in West Africa, specifically Guinea and Ghana, and the 2023 outbreaks in Equatorial Guinea and Tanzania emphasized the increase in frequency in the emergence of this high-consequence pathogen in new geographical regions expanding its endemic area [5,6,7,8]. The clinical manifestation of MVD progresses from initial non-specific flu-like symptoms to petechiae, delirium, multi-organ dysfunction, and hemorrhaging. The Syrian hamster model used in this study accurately recapitulates most hallmark signs of human MVD. Hamsters infected with hamster-adapted (HA-) MARV display fever, weight loss, and lethargy early on before severe signs of disease appear, such as coagulation abnormalities, hemorrhagic manifestations, petechial rash, and a severely dysregulated immune response [9]. There is neither an approved vaccine nor treatment for MVD, and due to the highly pathogenic nature and effective human-to-human transmission via bodily fluids, MARV is on the World Health Organization’s list of priority pathogens [10].

While there are no approved countermeasures available for MVD, several preclinical and clinical studies have been conducted bolstering the efforts for one to be made available. The most promising vaccine candidates thus far are based on recombinant vesicular stomatitis virus (VSV), adenovirus, and nucleic acid; the monoclonal antibody MR186YTE is a promising therapeutic in development [11,12,13,14,15,16]. To date, there have been clinical trials using the DNA and adenoviral vaccine platforms, demonstrating varying levels of immunogenicity [11,12,13]. In addition, VSV-MARV is currently evaluated in a phase 1 clinical trial (NCT06265012; www.clinicaltrials.gov; accessed on 20 June 2024). In this study, we sought to compare the protective efficacy of three vaccine platforms using a single dose of a VSV vector (live-attenuated), a non-replicating chimpanzee adenovirus vector (ChAdOx), and a LION™-formulated self-amplifying replicon RNA (repRNA) vaccine. The VSV-MARV vaccine expressing the MARV-Angola glycoprotein (GP) has previously demonstrated uniform protective efficacy after single-dose vaccination in non-human primate (NHP) studies, mainly mediated by humoral responses [14,17]. Furthermore, NHPs were protected when the vaccine was administered in as little as 7 days prior to challenge at a low dose, demonstrating the fast-acting and dose-sparing potential of this VSV-MARV vaccine [15]. The utility of the ChAdOx vaccine platform was demonstrated when it was deployed rapidly to combat the SARS-CoV-2 pandemic. The platform has been shown to elicit a balanced immune response, incorporating both humoral and cellular immunity to ensure the longevity of the induced protection [18,19]. The ChAdOx-MARV was generated following the successful example of the ChAdOx-nCov2019 vaccine and expresses the MARV-Angola GP. The final vaccine platform, repRNA/LION, based on a modified Venezuelan equine encephalitis virus RNA, is used to amplify protein-encoding mRNA of the target antigen. This method results in the expression of large amounts of antigen from fewer copies of RNA for a longer period of time [20,21]. While lipid nanoparticles are widely used for the RNA delivery mechanism [22], including repRNA, their application for the latter modality in humans has resulted in a dose-limiting reactogenic safety profile [23,24]. In contrast to traditional lipid nanoparticles that encapsulate RNA, LION electrostatically interacts with RNA molecules at the surface of the nanoparticles and exhibits a distinct localized biodistribution resulting in an improved safety profile [25]. The repRNA/LION platform has previously demonstrated robust antigen-specific humoral and cellular responses, offering a balanced immune response for protective correlates of protection [26,27,28,29]. A newly developed LION vaccine expressing the MARV-Angola GP (LION-MARV) was assessed for efficacy for the first time in this study. It is uncommon to directly compare multiple vaccine platforms in a single study as every developer focuses on their own product. However, a direct comparison of platforms enables not only protective efficacy comparison but also gives an opportunity to decipher the differences in the immune correlates of protection, which is normally performed retrospectively. 

## 2. Results

### 2.1. Single-Dose Vaccination with LION-MARV or VSV-MARV Uniformly Protects Hamsters from Lethal MARV Infection

**Figure 1:**
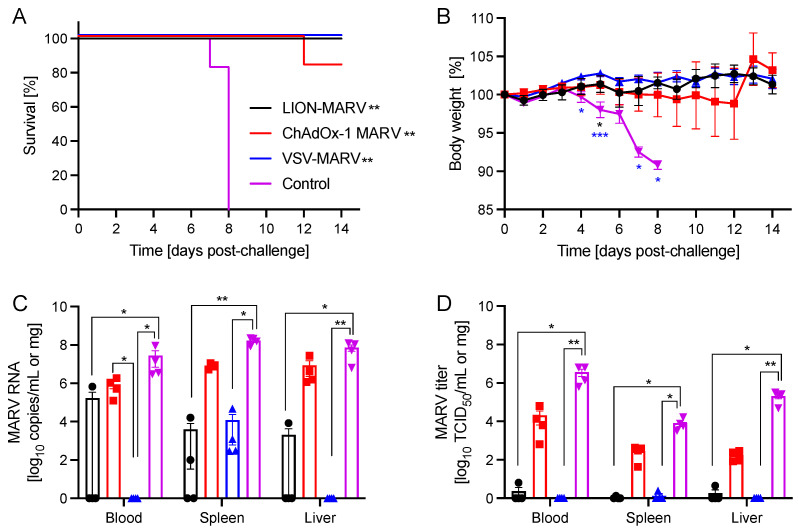
**VSV-MARV and LION-MARV completely protect hamsters from lethal challenge**. Hamsters were vaccinated with a single intramuscular dose of VSV-MARV, LION-MARV, ChAdOx-MARV, or kept naïve (Control). (**A**) Survival and (**B**) body weight changes during the acute phase of the disease are shown. MARV viremia and tissue viral loads were assessed by (**C**) RT-qPCR and (**D**) viral titration. Geometric mean and geometric SD are depicted in (**C**,**D**). Statistical significance of survival was determined by Mantel–Cox test, other data were evaluated by Kruskal–Wallis test with Dunn’s multiple comparisons. Statistical significance is indicated where achieved as *p* < 0.001 (***), *p* < 0.01 (**), and *p* < 0.05 (*). TCID_50_, median tissue culture infectious dose.

**Figure 2:**
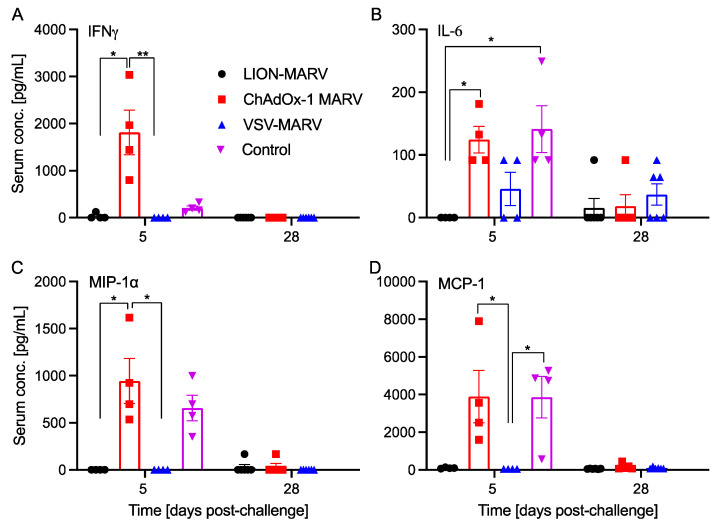
**ChAdOx-MARV-vaccinated hamsters demonstrate a protective and pathogenic cytokine profile**. Groups of hamsters were vaccinated with a single dose of VSV-MARV, LION-MARV, ChAdOx-MARV, or kept naïve (Control). The serum profiles of selected hamster cytokines including (**A**) IFNγ, (**B**) IL-6, (**C**) MIP-1α and (**D**) MCP-1 were assessed in the acute (day 5) and convalescent (day 28) phase of disease. Mean and standard error of the mean are depicted. Statistical significance was evaluated by Kruskal–Wallis test with Dunn’s multiple comparisons. Statistical significance is indicated where achieved as *p* < 0.01 (**), and *p* < 0.05 (*).

Syrian Golden hamsters (n = 10 per group) were vaccinated intramuscularly (IM) with a single dose of either 2 × 10^8^ infectious units (IU) of ChAdOx-MARV, 1 × 10^4^ plaque-forming-units (PFU) of VSV-MARV, or 10 μg of LION-MARV (all doses are standard doses for a small rodent model) 28 days prior to challenge. A group of naïve hamsters was used as controls. The challenge was performed with a lethal dose of HA-MARV by the intraperitoneal (IP) route on day 0. Naïve hamsters started to lose weight and showed other signs of disease starting at 4 days post-challenge (DPC), with all hamsters succumbing to disease 7–8 DPC. A single hamster in the ChAdOx1 cohort demonstrated weight loss starting at 8 DPC and succumbed to disease 12 DPC. All other hamsters vaccinated with ChAdOx-MARV displayed minimal weight loss and signs of disease. Notably, hamsters vaccinated with VSV-MARV and LION-MARV displayed no weight loss or signs of disease (Figure 1A,B). Investigation into the HA-MARV replication at 5 DPC demonstrated that the hamsters vaccinated with VSV-MARV had only low viral RNA levels in the spleen of all four hamsters, with no virus isolated from any of the collected samples (Figure 1C,D). LION-MARV-vaccinated hamsters had significantly reduced viremia and viral deposition in the liver and spleen compared to the control group. Viral RNA and titer were determined in some samples but not consistently (Figure 1). In contrast, samples from ChAdOx-MARV-vaccinated hamsters showed no significant decrease in viremia or tissue viral loads compared to the control cohort (Figure 1C,D). Interestingly, despite no apparent decrease in viral replication, 83% of the ChAdOx-MARV-vaccinated hamsters survived the lethal challenge. The serum cytokine profile of the hamsters during the acute phase of disease (5 DPC) and convalescence (28 DPC) demonstrated that the ChAdOx-MARV-vaccinated hamsters generated an antiviral response during the acute phase of disease generally associated with activated T-cells (Figure 2A,C). These hamsters also presented with upregulated levels of pathogenic cytokines suggesting continuous active viral replication during the acute phase of disease (Figure 2B,D). 

### 2.2. Antigen-Specific Humoral Immune Responses Post-Challenge and Functionalities Depend on the Vaccine Platform

**Figure 3:**
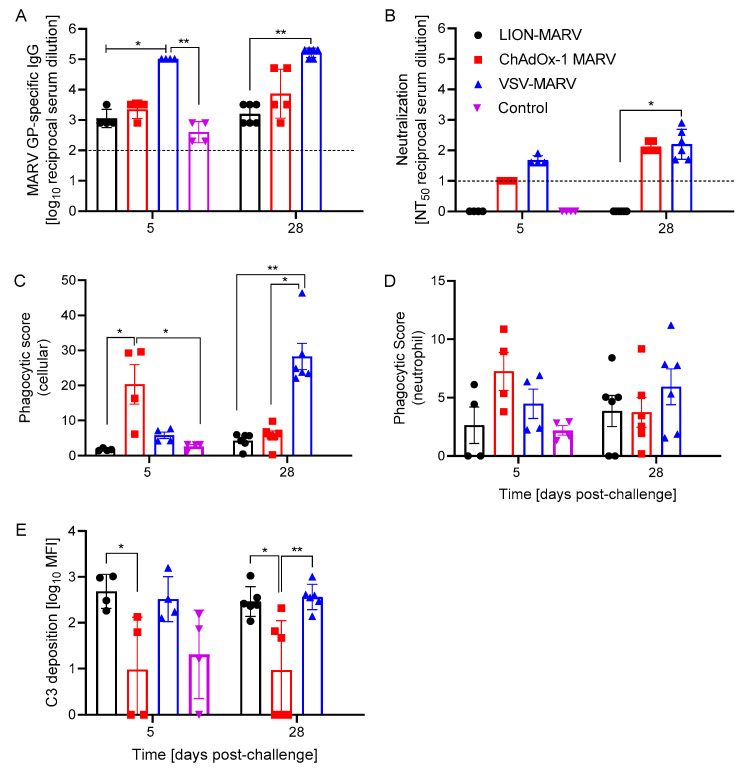
**Vaccine platform impacts the humoral response functionality profile.** (**A**) MARV GP-specific IgG titers in serum. Functionality of the antigen-specific humoral responses assessed by (**B**) 50% neutralization (NT_50_), (**C**) antibody-dependent cellular phagocytosis (ADCP), (**D**) antibody-dependent neutrophil phagocytosis (ADNP), and (**E**) antibody-dependent complement deposition (ADCD) in serum. Geometric mean and geometric SD are depicted in (**A**,**B**,**E**); mean with SEM is depicted in (**C**,**D**). Statistical significance was evaluated by Kruskal–Wallis test with Dunn’s multiple comparisons. Statistical significance is indicated where achieved as *p* < 0.01 (**) and *p* < 0.05 (*).

Next, we assessed the antigen-specific humoral response and its functionality to determine potential differences in the mechanism of action depending on the vaccine platform used. We first investigated the magnitude of the MARV GP-specific IgG antibody response. VSV-MARV-vaccinated hamsters presented with significantly higher GP-specific antibody titers 5 DPC compared to the other groups (Figure 3A). Interestingly, there remained a significant difference between the VSV-MARV antibody titers and that of the LION-MARV group 28 DPC (Figure 3A). These data suggest that the VSV vaccine produces the most robust binding antibody response and protection, with the LION vaccine also affording humoral protection, while the ChAdOx vaccine may be mediating protection predominantly by T cells. For the investigation of the functionality of the stimulated antibody response, we first assessed the neutralization potential using a VSV-MARV-GFP surrogate neutralization assay. In contrast to the MARV GP-specific ELISA, there were no significant differences at 5 DPC between the groups; however, only the VSV-MARV-vaccinated hamsters had detectable levels of neutralizing activity at 5 DPC. The only significant change was observed on 28 DPC between the VSV-MARV and LION-MARV hamsters (Figure 3B). Albeit not significant, the increase in the neutralizing titer in the ChAdOx-MARV group at 28 DPC indicates a boost effect of the challenge virus in vaccinated hamsters (Figure 3B). Next, we evaluated Fc effector functions, namely antibody-dependent cellular phagocytosis (ADCP), antibody-dependent neutrophil phagocytosis (ADNP), and antibody-dependent complement deposition (ADCD). The ChAdOx-MARV-vaccinated hamsters developed robust ADCP by 5 DPC. Antibody maturation resulted in the VSV-MARV-vaccinated hamsters showing significantly higher ADCP activity at the study endpoint (Figure 3C). However, the differences in ADCP between the vaccine platforms were not extended to the more specific ADNP assay (Figure 3D). The final Fc effector function, ADCD, demonstrated that at 5 DPC the LION-MARV-vaccinated hamsters demonstrated significantly higher C3 deposition levels compared to the ChAdOx-MARV-vaccinated hamsters; however, there were no significant differences between any of the vaccinated groups and the control hamsters. Antibody maturation revealed significantly lower C3 deposition in the ChAdOx-MARV-vaccinated hamsters compared to the VSV-MARV- or LION-MARV-vaccinated hamsters at 28 DPC (Figure 3E). These results indicate that the antibody functionality profile is dictated by the vaccine platform the antigen is expressed from. 

## 3. Discussion

In this study, we assessed the efficacy of single-dose vaccination with the VSV-MARV, LION-MARV, and ChAdOx-MARV administered 28 days prior to lethal HA-MARV challenge in Syrian Golden hamsters. Previous data have demonstrated single-dose protection with the VSV-MARV in NHPs, so it was no surprise that the hamsters were uniformly protected from lethality and disease [15]. However, this is one of the first studies investigating single-dose protection with the ChAdOx and repRNA/LION vaccines against filovirus infection. All hamsters receiving the VSV-MARV and LION-MARV vaccines were completely protected from lethality and disease. In contrast, only 83% of ChAdOx-MARV-vaccinated hamsters were protected from lethality. Surprisingly, the ChAdOx-MARV vaccine provided limited control of MARV replication in the blood and tissues of hamsters collected at 5 DPC, while the VSV-MARV and LION-MARV significantly reduced viral replication. This is unexpected, as the 83% survival rate of the ChAdOx-MARV group does not correlate, in its ratio, the reduced viral burden in the groups in which uniform protection is observed. We can only speculate that replication is controlled at a slower speed compared to these groups. 

It is known that the ChAdOx platform induces a strong humoral and cellular vaccine response [18,19]. Due to limited reagents in the hamster models, it is currently difficult to investigate cellular responses, in particular effector memory T-cell responses. This limitation and the time point used to assess viral replication may have skewed the data in favor of a sustained humoral response rather than a reactive cellular memory response. Following vaccination cellular responses peak 7–14 days post original antigenic exposure, after which 90–95% of the activated T-cells die off, leaving 5–10% of the memory T-cell population behind. Upon secondary antigenic exposure, memory T-cells can react and become activated within hours; but, due to the small cellular population that makes up memory T-cells, colonial expansion must take place to not only produce an activated cellular phenotype, but also have the cellular numbers necessary to induce a significant biological effect [30]. The memory T-cells have a delay in active replication 2–3 days post-secondary antigenic exposure; this would result in a window from viral challenge to robust cellular activation for MARV to replicate within the ChAdOx-MARV hamsters that may have resulted in a delay in viral clearance that we could not have seen at 5 DPC [31]. The potential contribution of T-cells was reaffirmed by a cytokine analysis, in which the ChAdOx-MARV-vaccinated hamsters displayed significantly higher amounts of IFNγ, which is indicative of a Th1 antiviral response. In contrast to the protective functions of IFNγ, ChAdOx-MARV-vaccinated hamsters also expressed significantly higher amounts of the pathogenic cytokines IL-6, MCP-1, and MIP-1α. IL-6 and MIP-1α have previously been demonstrated to be upregulated in the hamster MARV model [32]. MCP-1 has previously been associated with pathogenic functions and cytokine storm in other models of filovirus infections [15,33,34,35]. The cytokine data indicate that, at 5 DPC, the ChAdOx-MARV-vaccinated hamsters are mounting a protective immune response, possibly driven by the cellular immune response, while still experiencing pathogenic components with little effect on viral replication and inflammatory cytokine at the acute phase of disease.

The contribution of Fc effector functions of antibodies used to treat filovirus infections has been a double-edged sword. It has been previously demonstrated that Fc-mediated activation of natural killer cells and induction of phagocytic effector functions of immune cells have shown positive survival correlation [36,37]. There is also the potential for the Fc-mediated effector functions to induce enhanced disease by extrinsic and intrinsic mechanisms [38,39,40]. Historically, antibody responses have been shown to contribute to protection against viral diseases for all three vaccine platforms investigated in this study. The repRNA platform has been used in a number of preclinical vaccine studies; the most convincing of these is a study by Leventhal et al., who utilized B-cell-deficient mice or T-cell-depleted mice and demonstrated that B-cells are necessary for protection of mice from Crimean Congo hemorrhagic fever virus challenge [29]. The VSV platform has been specifically investigated for Fc effector functions for the VSV-MARV vaccination and challenge in cynomolgus macaques. Our group has previously demonstrated that VSV-MARV induces ADCD, ADCP, and ADCC [15]. Extensive work has been conducted on the ChAdOx-1 platform after its use to combat SARS-CoV-2. An investigation into the antibody effector functions has revealed that vaccination with ChAdOx can downregulate CD16 and reduce antibody-mediated NK cell activation [41]. Further investigation reconfirmed that ChAdOx lack an induction of ADCC and preferential activation of ADCP and ADCD when expressing a SARS-CoV-2 antigen [42]. The vaccine-platform-specific humoral response functionality demonstrated in other studies trended similarly in our study. We saw an early induction of ADCP at 5 DPC, and there was a trending increase in neutralization titer by 28 DPC demonstrating increased antibody maturation. VSV-MARV-vaccinated hamsters once again demonstrated ADCP and neutralization activity. We did see detectable levels of ADCD in this study, which is similar to the non-human primate data, but its magnitude did not increase the overtime in this study. The LION-MARV-vaccinated hamsters developed increased levels of ADCD, but limited to undetectable neutralization levels, which is reflected in previous studies [29]. The ability of the MARV GP to traffic efficiently to the plasma membrane may impact the production of binding and neutralizing antibodies; therefore, more work is needed to understand the biosynthesis of the repRNA-encoded MARV GP. Overall, the LION-MARV humoral response observed was lower compared to the other two platforms, specifically the VSV platform. This limited humoral response has been described before in other rodent studies using a single dose of the repRNA/LION platform, in which unique non-neutralizing antibodies and cellular responses were identified as the primary mechanism of protection [43]. Like the SARS-CoV-2 vaccine data, the ChAdOx-MARV-vaccinated hamsters developed a neutralizing and ADCP response but lacked the ADCD activity that was previously demonstrated. The differences we see in the functionality profiles may also be attributed to the antigen expressed by the platforms. Changes to the posttranslational modification, such as phosphorylation, glycosylation, and fucosylation of the antigen, will significantly alter the antibody functionality repertoire [44,45,46,47]. Overall, none of the vaccine platforms induced a significant amount of ADNP; however, the ChAdOx-MARV-vaccinated hamsters trended to have more at 5 DPC and the VSV-MARV-vaccinated hamsters trended to have more at 28 DPC. 

The limitations in this study include that only two time points were selected to investigate immune responses. The addition of more timepoints would allow for a greater breadth of understanding of antibody functionality early on; a later time point would enable us to determine if and when the ChAdOx-MARV-vaccinated hamsters control viral replication. Additionally, due to limited reagents available for hamsters, the cellular immune response was not assessed; specifically for the ChAdOx-MARV vaccine, this may be critical for vaccine efficacy. Finally, at this time, we do not have the ability to assess antibody-dependent cellular cytotoxicity, which is an additional Fc effector function that would be interesting to assess. 

In summation, we compared the protective efficacy and humoral responses of three vaccine platforms to combat lethal MARV challenge in hamsters. We determined that, when hamsters were vaccinated 28 days before challenge, the LION-MARV and VSV-MARV vaccines provided uniform protection, while only 83% of ChAdOx-MARV-vaccinated hamsters were protected. Systemic MARV replication was controlled by 5 DPC in the VSV-MARV- and LION-MARV-vaccinated hamsters, while the ChAdOx-MARV-vaccinated hamsters did not control viral replication at this time point. All three vaccine candidates induced an antigen-specific humoral response with the VSV-MARV-vaccinated hamsters, producing significantly more antigen-specific IgG. The Fc effector functionality profile of this response was vaccine-platform-dependent. Overall, each vaccine platform warrants further investigation in the NHP model, which is the gold standard for preclinical filovirus vaccine testing. 

## 4. Materials and Methods

### 4.1. Ethics Statement

All work involving MARV was performed in the maximum containment laboratory (MCL) at the Rocky Mountain Laboratories (RML), Division of Intramural Research, National Institute of Allergy and Infectious Diseases, National Institutes of Health. RML is an Association for Assessment and Accreditation of Laboratory Animal Care International (AAALAC)-accredited institution. All procedures followed RML Institutional Biosafety Committee (IBC)-approved standard operating procedures (SOPs). Animal work was performed in strict accordance with the recommendations described in the Guide for the Care and Use of Laboratory Animals of the National Institute of Health, the Office of Animal Welfare, and the Animal Welfare Act, United States Department of Agriculture. This study was approved by the RML Animal Care and Use Committee (ACUC), and all procedures were conducted on anesthetized hamsters by trained personnel under the supervision of board-certified clinical veterinarians. The hamsters were observed at least twice daily for clinical signs of disease according to an RML ACUC-approved scoring sheet and they were humanely euthanized when they reached the endpoint criteria. 

### 4.2. Cells and Viruses

Vero E6 cells (*Mycoplasma* negative) were grown at 37 °C and 5% CO_2_ in Dulbecco’s modified Eagle’s medium (DMEM) (Sigma-Aldrich, St. Louis, MO, USA) containing 10% fetal bovine serum (FBS) (Wisent Inc., St. Bruno, QC, Canada), 2 mM L-glutamine, 50 U/mL penicillin, and 50 μg/mL streptomycin (all supplements from Thermo Fisher Scientific, Waltham, MA, USA). THP-1 (*Mycoplasma* negative) were grown at 37 °C and 5% CO_2_ in Roswell Park Memorial Institute (RPMI) medium (Sigma-Aldrich, St. Louis, MO, USA) containing 10% FBS, 2 mM L-glutamine, 50 U/mL penicillin, and 50 μg/mL streptomycin. HL-60 cells (ATCC; Cat. No. CCL-240) were propagated in Iscove’s Modified Dulbecco’s medium (IMDM) with 20% FBS, 2 mM L-glutamine, 50 U/mL penicillin, and 50 mg/mL streptomycin and differentiated with 1.3% DMSO for 5 days. For the hamster challenge, HA-MARV, based on MARV-Angola, at 100 LD_50_ was used IP as previously described [32].

### 4.3. Vaccines

The VSV-MARV is based on the VSVΔG platform, presenting a live-attenuated vaccine expressing the MARV-Angola GP and was generated as described previously [48]. The same product as described in [49] was used for this study. Secondly, LION-MARV vaccine was prepared as previously described [26] using the same lot of a cationic nanocarrier (LION) complexed with the same lot of an alphavirus-derived replicating RNA (repRNA) encoding the full-length GP of MARV variant Angola. Third, MARV variant Angola GP was codon optimized for humans and synthesized by GeneArt (Thermo Fisher Scientific). The synthesized GP gene was cloned into a transgene expression plasmid, comprising a modified human cytomegalovirus immediate early promoter (CMV promoter) with tetracycline operator sites and the polyadenylation signal from bovine growth hormone. The resulting expression cassette was inserted into the E1 locus of a genomic clone of ChAdOx1 using site-specific recombination [50]. The virus was recovered and propagated in T-Rex 293 cells (Invitrogen) and purified by CsCl gradient ultracentrifugation. Virus titration was performed as previously described [51,52].

### 4.4. Study Design

Forty male Syrian Golden hamsters (*Mesocricetus auratus*) were randomly divided into groups of 10 hamsters and were vaccinated IM with a single dose of 2 × 10^8^ IU of the ChAdOx1 vaccine, 1 × 10^4^ PFU of VSV-MARV, or 10 μg of the LION-MARV 28 days prior to challenge. The fourth group of hamsters remained naïve. The challenge was performed with a lethal dose of 100 LD_50_ (1 PFU) of HA-MARV IP on day 0. Hamster cohorts for sample analysis were euthanized on 5 DPC which corresponds to peak viremia as described previously [32]. Hamsters were observed at least twice daily for clinical signs of disease. Body weight was measured once a day, and animals were humanely euthanized when they reached RML ACUC-approved endpoint criteria. Surviving hamsters were kept until 28 DPC when a terminal serum sample was collected for analysis. 

### 4.5. Viral Load Quantification

RT-qPCR assays were performed on RNA extracted for blood samples using the QIAamp Viral RNA Mini Kit (Qiagen, Hilden, Germany) according to manufacturer specifications. For tissue samples, a maximum of 30 mg each, were processed and extracted using the RNeasy Mini Kit (Qiagen) according to manufacturer specifications. One-step RT-qPCR for genomic viral RNA was performed using specific primer-probe sets to MARV L gene, and the QuantiFast Probe RT-PCR +ROX Vial Kit (Qiagen), in the Rotor-Gene Q (Qiagen), as previously described [15]. Infectious viral titers were determined from EDTA whole blood or tissue homogenates using Vero E6 cells (*Mycoplasma* negative) as previously described. The 50% tissue culture infectious dose (TCID_50_) was calculated for each sample using the Reed and Muench method [53].

### 4.6. Cytokine Analysis

Post-challenge hamster serum samples were inactivated by γ-irradiation (4 MRad), a well-established method with minimal impact on serum antibody binding [54] and removed from the MCL according to SOPs approved by the RML IBC. 

Serum cytokine analysis was performed for 5 DPC and 28 DPC on serum samples. Hamster serum samples were diluted 1:5 as per manufacturer’s instructions and assessed using the Ampersand Biosciences 9-plex hamster cytokine panel (Lake Clear, NY, USA). Values for IL-10, IL-2, IL-4, IL-6, IFNγ, MCP-1, MIP-1α, TNFα, and VEGF were assessed. Values below the limit of detection of the assay were assigned the value of 0. 

### 4.7. Assessment of Humoral Immune Response

ELISAs were conducted as previously described [15]. Briefly, serum samples were inactivated by γ-irradiation and used in BSL2 according to RML IBC-approved SOPs. ELISA plates were coated with 1 µg/mL of recombinant MARV-Angola GPΔTM (IBT Bioservices, Rockville, MD, USA). The plates were washed 3 times with PBS/Tween and blocked with 5% milk buffer. The serum samples were diluted 1:100 and 4-fold serial dilutions thereafter. A secondary antibody specific to hamster was used to assess IgG (Abcam, Cambridge, UK). The optical density at 405 nm was measured using a GloMAx explorer (BioRad, Hercules, CA, USA). Baseline samples obtained from naïve hamsters were used to determine the cutoff value (mean OD plus three times the standard deviation). Neutralization assays with VSV-MARV-GFP were performed, as previously described [55]. The VSV-MARV-GFP assay was optimized, and incubation of serum dilution mix on the Vero E6 cells lasted for 16 h at 37 °C. Samples were run on the FACSymphony A5 Cell Analyzer (BD Biosciences, Mississauga, ON, Canada) and the GFP-positive cell count was determined.

### 4.8. Quantification of Antibody Effector Functions

Assays for antibody effector functions were adapted from previously established protocols [15,56]. Recombinant MARV GPΔTM (IBT Bioservices) was tethered to Fluospheres NutrAvidin-Microspheres yellow–green or red (Thermo Fisher Scientific, Waltham, MA, USA) using the EZ-link Micro Sulfo-NHS-LC-Biotinylation kit (Thermo Fisher Scientific). 

### 4.9. Antibody-Dependent Complement Deposition (ADCD)

Serum samples were heat-inactivated at 56 °C for 30 min then diluted 1:100 in DMEM and applied to the conjugated beads (20 μL/well) for one hour at 37 °C. Next, guinea pig complement (Cedarlane, Burlington, ON, Canada) was added for 30 min. The bead complexes were washed with PBS containing 15 mM EDTA and stained with anti-C3c-FITC (Antibodies-Online, Cat. No. ABIN458597). Data were acquired on a FACS Symphony (BD, Franklin Lakes, NJ, USA) and analyzed in FlowJo v10. 

### 4.10. Antibody-Dependent Neutrophil Phagocytosis (ADNP)

Biotinylated MARV GPΔTM was coupled to yellow green Neutravidin beads (Life Technologies, Carlsbad, CA, USA). Serum samples were diluted 1:100 in culture medium and incubated with GP-coated beads for 2 h at 37 °C. Beads (20 μL) were added to HL-60 cells (5 × 10^4^ cells/well) and incubated for 2 h at 37 °C. Cells were then stained for CD11b (Clone G10F5; BioLegend, San Diego, CA, USA), CD16 (Clone UCHT1; BD Biosciences), and fixed with 4% paraformaldehyde. Data were acquired on a FACS Symphony (BD) and analyzed in FlowJo v10. Neutrophils were defined as SSC-A^high^ CD11b^+^, CD16+. A phagocytic score was determined using the following formula: (percentage of FITC+ cells) × (geometric mean fluorescent intensity (gMFI) of the FITC+ cells)/10,000. 

### 4.11. Antibody-Dependent Cellular Phagocytosis (ADCP)

Serum samples were diluted 1:100 in DMEM and incubated with 20 μL of the conjugated beads for 2 h at 37 °C. The serum/bead mixture was then transferred to a plate of THP-1 cells (2.5 × 10^4^ cells/well) and incubated overnight at 37 °C. Cells were fixed with 4% paraformaldehyde. Data were acquired on a FACS Symphony (BD) and analyzed in FlowJo v10. A phagocytic score was determined using the following formula: (percentage of FITC+ cells) × (geometric mean fluorescent intensity (gMFI) of the FITC+ cells)/10,000.

### 4.12. Statistical Analysis

All statistical analysis was performed in Prism 9 (GraphPad). Statistical significance of survival was determined by log/rank Mantel–Cox test. All other data were evaluated by Kruskal–Wallis test with Dunn’s multiple comparisons. Statistical significance was achieved at *p* < 0.05 and is indicated in each figure. 

## Data Availability

The original data presented in the study are included in the article; further inquiries can be directed to the corresponding author.
